# Reasons for Undesirable Pregnancy Outcomes among Women with Appendicitis: The Experience of a Tertiary Center

**DOI:** 10.1155/2020/6039862

**Published:** 2020-09-15

**Authors:** Baris Mantoglu, Fatih Altintoprak, Necattin Firat, Emre Gonullu, Enis Dikicier, Yesim Akdeniz, Mehmet Aziret, Unal Erkorkmaz

**Affiliations:** ^1^Sakarya University Educating and Research Hospital, Department of General Surgery, Serdivan, Sakarya, Turkey; ^2^Sakarya University, Faculty of Medicine, Department of General Surgery, Serdivan, Sakarya, Turkey; ^3^Sakarya University Educating and Research Hospital, Department of Gastroenterological Surgery, Serdivan, Sakarya, Turkey; ^4^Sakarya University, Faculty of Medicine, Department of Biostatistics, Serdivan, Sakarya, Turkey

## Abstract

**Background:**

Although laparoscopic appendectomy increases its popularity today, the answer to the question of whether to perform open or laparoscopic appendectomy during pregnancy is appropriate in many studies, and the choice of surgery depends on the surgeon. Herein, we aimed to evaluate the variables that affect undesirable pregnancy outcomes that occur as a result of appendicitis during pregnancy.

**Methods:**

Seventy-eight pregnant patients with acute appendicitis who underwent laparoscopic or open technique intervention enrolled in this retrospective study. In addition to the demographic structure of the patients, surgical technique, the number of pregnancies, multiple pregnancy status, surgical pathologies, laboratory values, radiological imaging methods, and length of hospital stay were evaluated. The severity of appendicitis was classified according to the pathology results. The patients were divided into two groups according to the outcomes of their pregnancy. Preterm delivery and abortion involved in the study as a single complication section.

**Results:**

The mean age of the pregnant patients was 28.6 ± 5. Of the 78 pregnant women with appendicitis, 47.4% had their first pregnancy, 37.2% had their second pregnancy, and 15.4% had 3 or more pregnancies. The preterm delivery and abortus were 19.5% in the open appendectomy (OA) group and 16.2% in the laparoscopic appendectomy (LA) group. No statistically significant difference was detected in this group in terms of appendicitis pathology triggering preterm delivery or abortion (*p* 0.075). When white blood count (WBC) and C-reactive protein (CRP) were evaluated by laboratory findings, CRP was found to be statistically significantly higher in patients with preterm birth (*p* 0.042).

**Conclusion:**

Consequently, acute appendicitis may cause serious intra-abdominal infection and inflammation in addition to the complexity of the diagnosis due to the nature of pregnancy, as well as undesired pregnancy outcomes with the surgical technique, or independently with other variables.

## 1. Introduction

Acute appendicitis is the most common emergency condition seen in general surgery practice and the most common cause of a nonobstetric surgical emergency during pregnancy. The incidence of acute appendicitis in pregnant women (1.8–41 per 10,000 pregnancies) is not different from that in nonpregnant female patients of the same age [1, 2]. Although acute appendicitis can occur during all trimesters, it is most frequent in the second trimester [3–5]. Abdominal surgical procedures, particularly, appendectomy during pregnancy, can lead to poor pregnancy outcomes [6]. Appendicitis is challenging to treat during pregnancy due to leukocytosis, displacement of the appendix, and the limited applicability of imaging techniques. A delay in diagnosis and treatment results in more complicated appendicitis and an increased likelihood of preterm labor, perinatal morbidity, mortality, and fetal loss [6–10].

Appendicitis surgery during pregnancy can be performed via an open or laparoscopic method according to current guidelines [11, 12]. It remains unclear as to which surgical technique is superior during pregnancy. Although a recent meta-analysis reported that laparoscopic appendectomy (LA) and open appendectomy (OA) groups did not differ in terms of the rate of preterm labor, OA was not safer than LA in terms of pregnancy outcomes, and LA was superior in terms of surgical outcomes [13].

In this study, we evaluated the associations of the number of pregnancies, parity, time to hospital admission, surgical technique, and laboratory parameters with undesirable pregnancy outcomes among patients diagnosed with appendicitis.

## 2. Methods

This retrospective study included 78 pregnant patients diagnosed with appendicitis, among 606 women of reproductive age with acute appendicitis admitted to the Sakarya University Training and Research Hospital Emergency Department between May 2014 and May 2019. Patient demographics, surgical technique, number of pregnancies, multiple pregnancy status, surgical pathologies, laboratory data, radiological imaging methods, and length of hospital stay were evaluated. The severity of appendicitis was classified according to the pathology results. The patients were divided into two groups according to pregnancy outcome. The first group consisted of 64 patients whose pregnancy was free from adverse events (healthy birth at term), whereas the second group consisted of 14 patients with undesirable pregnancy outcomes (abortion or preterm labor). Preterm labor and abortions were considered together due to the low number of such cases. Along with the operative technique, the number of pregnancies and differences according to trimester was examined. We also determined whether the number of pregnancies was associated with time to hospital admission and the rate of preterm labor/abortion. Conventional appendectomies were more frequent than LA during the first few years of the study, but the number of patients undergoing LA increased in subsequent years. This was due to changes in surgeon preferences and an increase in experience and the number of viable surgical techniques. All patients were checked before and after surgery by an obstetrician, and all patients were provided with tocolytic agents as prophylactic treatment to prevent postoperative uterine contractions. The study was approved by the ethics committee of our university (71522473/050.01.04/83).

### 2.1. Statistical Analysis

Descriptive analyses were performed on the general characteristics of the study population. The Kolmogorov–Smirnov test was used to evaluate the normality of the numerical data. The Kruskal–Wallis test was used to compare numerical variables among three groups. The Mann–Whitney *U* test was used to compare the numerical variables between two groups. The numerical variables are presented as medians (interquartile range). Categorical variables were compared with the chi-square test and are presented as counts and percentages. A *p* value <0.05 was considered significant. Analyses were performed using SPSS statistical software (version 23.0; IBM Corp., Armonk, NY, USA).

## 3. Results

The mean age of the pregnant patients was 28.6 ± 5 years. Of the 78 pregnant women with appendicitis, 47.4% had their first pregnancy, 37.2% had their second pregnancy, and 15.4% had 3 or more pregnancies. About 95% of our patients had single pregnancies; 3.8% had twin pregnancies, and 1.3% triplet pregnancies. About 32% of the patients had single pregnancy, while 42.3% two pregnancies and 25.6% three or more pregnancies. Abdominal pain was a symptom in all patients, while nausea and vomiting were detected in 48% and 26%, respectively. About 76% of the patients were referred to the hospital within 24 hours of the onset of abdominal pain, while 16.7% were referred within 2 days and 7.7% on day 3 (Table 1). The hospital admission rate on the first day of abdominal pain was 78.4% for the single parity patients and 73.2% for those with two or more parities (Table 2). The mean hospital stay was 1 day in the LA group and 2 days in the OA group (*p*=0.348).

Preterm status was divided into three periods according to the time (number of gestational weeks) between appendectomy and preterm labor. Preterm labor occurred at a mean of 10.4 ± 5.381 weeks after appendectomy. In about 50% of cases, preterm labor occurred between 31 and 35 weeks. All abortions were detected just before or immediately after an appendectomy; 50% of the abortions occurred during the first trimester and 50% during the second trimester (Table 3).

Abdominal ultrasonography (USG) was performed in 98.7% of the patients at the time of diagnosis. Appendicitis was diagnosed in 71.8% of the patients. Magnetic resonance imaging (MRI) was performed in 22 patients not diagnosed using USG. The diagnosis was clarified by MRI in 54.5% of the patients who could not be otherwise diagnosed (Table 1).

About 53% of our patients underwent OA, and 47.4% underwent LA. About 82% of the patients were operated under general anesthesia and 17.9% were operated under spinal anesthesia. Complications related to the surgical intervention developed in 2.6% of patients; one had a surgical site infection, and another had a 3-day headache due to spinal anesthesia. About 82% of our pregnant patients had term deliveries. The percentage of preterm delivery/abortion was 19.5% in the OA group and 16.2% in the LA group (*p*=0.934) (Table 4).

In total, 83.9% of the patients diagnosed with acute appendicitis had a term birth, 10.7% had preterm labor, and 5.4% had an abortion. About 56% of patients who had a perforated appendix delivered at term, 33.3% had preterm labor, and 11% had an abortion. Lymphoid hyperplasia determined our negative appendectomy rate of 16.7%. In total, 92.3% of patients with lymphoid hyperplasia had a term delivery; the remaining 7.7% had preterm labor, and there were no abortions. The rate of preterm delivery/abortion did not differ between the perforated appendix and lymphoid hyperplasia patients (*p*=0.075) (Table 4).

The preoperative white blood cell (WBC) count was 12.8 (5.75) × 10^3^ mm^3^ in the term group and 14.6 (6.35) × 10^3^ mm^3^ in the preterm/abortion group. The C-reactive protein (CRP) level was 16.5 (46.54) mg/L in the term group and 78.5 (110.55) mg/L in the preterm/abortion group (*p*=0.042) (Table 5).

In total, 83.8% of the first-pregnancy patients delivered at term, while preterm labor/abortion occurred in 16.2% of these patients. The rate of birth at term was 80.5% in patients with two or more pregnancies; thus, the preterm labor/abortion rate was 19.5% (*p*=0.934) (Table 4). Moreover, there were no significant differences in undesirable single or multiple pregnancy outcomes according to the surgical technique (*p*=0.146) (Table 4).

The rate of adverse pregnancy outcomes did not differ among surgical techniques in any trimester (Table 6). Similarly, the number of cases of preterm labor/abortion did not differ by the surgical technique (Table 6).

## 4. Discussion

Appendicitis during pregnancy is a challenging medical condition that has undesirable consequences with a delay in the diagnosis, which is more challenging than in nonpregnant individuals. The difficulty in diagnosis is due to symptoms that overlap with those that occur normally during pregnancy, such as nausea and vomiting, as well as to the changes in laboratory findings that characterize normal pregnancy [14–16]. No significant differences were observed between our patients without adverse events and those with undesirable pregnancy outcomes in terms of symptoms or clinical examination results.

Early accurate diagnosis of appendicitis is crucial to prevent maternal and fetal morbidity and mortality and unnecessary surgical intervention [17]. The rate of negative appendectomy according to our retrospective analysis was 16.6%, which is lower than those reported previously [1, 18]. The limited utility of imaging modalities during pregnancy also complicates the diagnosis of appendicitis. The appendix is assumed to be displaced by the expanding uterus during pregnancy, with the extent of displacement depending on the gestational week [19, 20]. However, according to one study, this is the only accepted dogma and not actually the case [21, 22]. USG is the most useful radiological imaging modality for diagnosing appendicitis during pregnancy; however, we also used MRI in patients who could not be diagnosed based on USG. In total, 12.8% of cases could not be diagnosed radiologically.

According to guidelines published by the Society of American Gastrointestinal and Endoscopic Surgeons, LA can be used safely in pregnant women. Some meta-analyses and reviews have suggested that there is insufficient evidence regarding the safety of laparoscopy during pregnancy; indeed, it has been reported to cause fetal loss [23, 24]. A recent meta-analysis of surgical techniques confirmed that obstetric outcomes do not differ between LA and OA [13]. Among our patients, there was no significant difference in terms of obstetric outcomes between the LA and OA groups during any of the three trimesters.

As indicated in many studies, serum WBC and CRP levels increase in patients with acute appendicitis, and the laparoscopic method is recommended [25]. CRP values are lower during the postoperative period with the laparoscopic versus open method, and postoperative recovery is also better with the former method [25]. The length of hospital stay was 1 day in patients who underwent LA and 2 days in those who received OA. This shorter hospital stay has been highlighted in several studies as the most significant benefit of laparoscopic surgery [25, 26]. In our study, the preoperative CRP level was significantly higher in the preterm labor/abortion group compared with the group free from adverse events. However, the number of patients was insufficient to perform a multivariate analysis and thus draws any definitive conclusion. Perforated appendix increases the likelihood of fetal loss compared with uncomplicated appendicitis [27]. Preterm labor or miscarriage occurred in four of our nine perforated appendicitis patients, but statistical analysis could not be performed due to the limited number of patients (Table 2). As reported in a previous study, infection and inflammation lead to undesirable fetal outcomes regardless of the surgical technique used; our findings support this [28]. As noted in Borst's study, perforated appendix was more frequent in pregnant women than in nonpregnant individuals [29]. Early admission and diagnosis prevent adverse outcomes in pregnant women with appendicitis. In women who have had multiple pregnancies, hospital admission may be delayed, which may in turn lead to a delay in the diagnosis of appendicitis. Any delay can increase the likelihood of progression to an infectious inflammatory state, which could lead to perforated appendix. An increase in the CRP level may predict preterm delivery and fetal loss. Some studies have reported a lower pain threshold among primipara mothers compared with multipara mothers [30, 31]. In our study, the single- and multigravid patients were not different in terms of time to hospital admission (Table 4). Moreover, neither the number of pregnancies nor the time to hospital admission was associated with undesirable pregnancy outcomes after OA or LA (Table 6).

In addition to its retrospective nature, the major limitation of this study was the small number of cases included due to the low incidence of appendicitis during pregnancy. Nevertheless, the association of time to hospital admission with the pregnancy outcomes of patients diagnosed with appendicitis has not been evaluated before. Inflammatory markers may be important for predicting preterm labor and abortion. Our data should serve as an important resource for future studies.

In conclusion, many factors can cause undesirable pregnancy outcomes in women with appendicitis. Inflammation and infection should not be ignored and may indicate the need for surgery.

## Figures and Tables

**Table 1:** Descriptive statistics of categorical variables.

Variables		Number (percent)
Pregnancy	1	37 (47.4%)
	2	29 (37.2%)
	3	5 (6.4%)
	4	5 (6.4%)
	5	1 (1.3%)
	8	1 (1.3%)

Total number of pregnancies	1	25 (32.1%)
	2	33 (42.3%)
	3	10 (12.8%)
	4	5 (6.4%)
	5	3 (3.8%)
	6	1 (1.3%)
	8	1 (1.3%)

Multiple pregnancy	Single	74 (94.9%)
	Twin	3 (3.8%)
	Triplet	1 (1.3%)

Nausea	No	48 (61.5%)
	Yes	30 (38.5%)

Vomiting	No	52 (66.7%)
	Yes	26 (33.3%)

Abdominal pain	Yes	78 (100%)

Hospital admission time interval	1 day	59 (75.6%)
	2 days	13 (16.7%)
	3 days	6 (7.7%)

USG^*∗*^	None	1 (1.3%)
	Yes	77 (98.7%)

MRI^*∗∗*^	None	56 (71.8%)
	Yes	22 (28.2%)
	Total	78 (100%)

DUSG^*∗∗∗*^	Negative	22 (28,2%)
	Positive	56 (71.8%)

DMR^*∗∗∗∗*^	Negative	10 (45.5%)
	Positive	12 (54.5%)
Type of surgery	Open	41 (52.6%)
	Laparoscopic	37 (47.4%)
	Total	78 (100%)

Type of anesthesia	General	64 (82.1%)
	Spinal	14 (17.9%)

Complication	None	76 (97.4%)
	Headache due to spinal	1 (1.3%)
	Surgical site infection	1 (1.3%)

Birth	Term	64 (82.1%)
	Preterm	10 (12.8%)
	Abortus	4 (5.1%)

^*∗*^Abdominal ultrasonography; ^*∗∗*^magnetic resonance imaging; ^*∗∗∗*^diagnostic ultrasonography; ^*∗∗∗∗*^diagnostic magnetic resonance imaging.

**Table 2:** Relationship between number of pregnancies and admission period.

		Admission time			*p* value	Effect size
		1	2	3		
Pregnancy	1	29 (78.4%)	5 (13.5%)	3 (8.1%)	0.855	0.080
	2+	30 (73.2%)	8 (19.5%)	3 (7.3%)		

**Table 3:** Relationship between the appendectomy week in pregnancy and the weeks of observing preterm-abortion status.

Week of appendectomy	Preterm 25–30 w	Preterm 31–35 w	Preterm 36–39 w	Abortus
30 w	—	—	37 w	—
34 w	—	34 w	—	—
32 w	—	34 w	—	—
28 w	30 w	—	—	—
20 w	—	32 w	—	—
17 w	—	—	36 w	—
10 w	—	35 w	—	—
15 w	—	34 w	—	—
28 w	30 w	—	—	—
14 w	28 w	—	—	—
19 w	—	—	—	19 w
6 w	—	—	—	6 w
18 w	—	—	—	18 w
6 w	—	—	—	6 w

^*∗*^The mean preterm incidence after appendectomy is 10.4 ± 5.381 weeks; ^*∗*^50% of preterm labor observed between 31 and 35 weeks period; ^*∗*^all abortuses were detected just before or immediately after appendectomy; ^*∗*^50% of the abortions occurred in the 1st trimester and 50% in the 2nd trimester. ^*∗∗*^W: week.

**Table 4:** Relation of various states with delivery.

		Delivery		*p* value	Effect size
		Term	Preterm + abort		
Pathology	Acute appendicitis	47 (83.9%)	9 (16.1%)	0.075	0.262
	Perforated appendicitis	5 (55.6%)	4 (44.4%)		
	Lymphoid hyperplasia	12 (92.3%)	1 (7.7%)		

Surgery	Open	33 (80.5%)	8 (19.5%)	0.934	0.043
	Laparoscopic	31 (83.8%)	6 (16.2%)		

Pregnancy	First	31 (83.8%)	6 (16.2%)	0.934	0.043
	2+	33 (80.5%)	8 (19.5%)		

Multiple pregnancy	Single	62 (83.8%)	12 (16.2%)	0.146	0.258
	Twin	2 (66.7%)	1 (33.3%)		
	Triplet	0 (0%)	1 (100%)		

**Table 5:** Effects of WBC and CRP on delivery.

	Delivery		MW		
	Normal (*n* = 64)	Preterm + abort (*n* = 14)	*Z* value	*p* value	Effect size
WBC	12.8 [5.75]	14.6 [6.35]	−1.602	0.109	−0.181
CRP	16.5 [46.54]	78.5 [110.55]	−2.038	0.042	−0.231

**Table 6:** Relationship between the first pregnancy and multiple pregnancies and trimester-based surgical methods on delivery.

Surgery type		Delivery		*p* value	Effect size
		Term	Preterm + abort		
First pregnancy	Open	16 (80%)	4 (20%)	0.667	0.111
	Laparoscopic	15 (88.2%)	2 (11.8%)		
2+ pregnancy	Open	17 (81%)	4 (19%)	1.000	0.012
	Laparoscopic	16 (80%)	4 (20%)		
Trimester 1	Open	10 (83.3%)	2 (16.7%)	1.000	0.057
	Laparoscopic	7 (87.5%)	1 (12.5%)		
Trimester 2	Open	16 (88.9%)	2 (11.1%)	0.685	0.079
	Laparoscopic	20 (83.3%)	4 (16.7%)		
Trimester 3	Open	7 (63.6%)	4 (36.4%)	1.000	0.164
	Laparoscopic	4 (80%)	1 (20%)		

## Data Availability

The data used in the study were obtained from the archives of Sakarya University Medical Faculty, Educating and Research Hospital, General Surgery Clinic, after obtaining institutional and ethical approvals. The data used to support the findings of this study are available from the corresponding author upon request.
